# Introduction to this Special Issue

**DOI:** 10.1080/16549716.2021.1918913

**Published:** 2021-04-20

## Guest Editor

Ntombizodwa Ndlovu and Boyd Swinburn

## Contents

## Editorial

Readiness for sugar sweetened beverage taxation in sub-Saharan Africa

## Articles

Study design: policy landscape analysis for sugar-sweetened beverage taxation in seven sub-Saharan African countries

The political economy of sugar-sweetened beverage taxation: an analysis from seven countries in sub-Saharan Africa

The data availability landscape in seven sub-Saharan African countries and its role in strengthening sugar-sweetened beverage taxation

The legal feasibility of adopting a sugar-sweetened beverage tax in seven sub-Saharan African countries

Nutrition related non-communicable diseases and sugar sweetened beverage policies: a landscape analysis in Zambia

Barriers to, and facilitators to, the adoption of a sugar sweetened beverage tax to prevent non-communicable diseases in Namibia: a policy landscape analysis

Strengthening prevention of nutrition-related non-communicable diseases through fiscal policies in Rwanda: a policy landscape analysis

Nutrition related non-communicable disease and sugar sweetened beverage policies: a landscape analysis in Kenya

Barriers to, and facilitators to, the adoption of a sugar sweetened beverage tax to prevent non-communicable diseases in Uganda: a policy landscape analysis

## Dedication

Professor Peter Byass

3 April 1957 - 16 August 2020

This Special Issue of Global Health Action is dedicated to memory of Professor Peter Byass – a colleague, friend and mentor to many. Prior to his passing in 2020, Prof Byass WHO served as the Editor in Chief of Global Health Action, played a leading role in the conceptualization of this Special Issue. Prof Byass’s career as an epidemiologist centered on a commitment to promoting health research in low-and-middle income countries and growing the careers of many young researchers in these countries. His collaborative approach to research was a key part of his role as the founding leader of the INDEPTH Network, an epidemiological and demographic surveillance system which builds capacity among many early career researchers. Perhaps the most telling contribution Prof Byass made to the science community can be observed in the outpouring of moving words and dedications from his colleagues and students, many of whom are key figures in Global Health today. He was described as a “pioneer”, “mentor” and “friend”. In many ways, the contents of this Issue, which focuses on promoting health throughout Sub-Saharan Africa, builds on Peter’s vision of a healthier and more equitable global society. His loss is felt by the entire global health community. May he rest in peace.

## Introduction

Countries in sub-Saharan Africa are facing a growing burden of non-communicable diseases (NCDs). By 2030, it is estimated that NCDs will become the leading cause of death in the continent, overtaking HIV and tuberculosis. Interventions such as sugar-sweetened beverage (SSB) taxation have the potential to turn the tide of this epidemic. Many countries, including South Africa and Botswana, have adopted SSB taxes for this reason, although their initiatives have proved complex and challenging.

In 2017, the SAMRC Centre for Health Economics and Decision Science (PRICELESS SA) launched an ambitious multi-country study to assess readiness to adopt a sugary beverage tax for the prevention of NCDs in sub-Saharan Africa. The study took place in seven countries, Botswana, Kenya, Namibia, Rwanda, Tanzania, Uganda and Zambia, and involved over twelve researchers from nine different countries. For many, this was the first research to be undertaken on sugary beverage taxation, or even NCD policy.

This research provides a foundation for supporting future action on NCD prevention in sub-Saharan Africa. The study has culminated in this Special Issue of Global Health Action, “Readiness for Sugar Sweetened Beverage Taxation in Sub-Saharan Africa” guest edited by Boyd Swinburn and Zodwa Ndlovu.

We wish to acknowledge, with gratitude, the generous support of the International Development Research Centre, which made this research possible, as well as the hard work of the Global Health Action Editorial Team. In particular, we acknowledge the late Professor Peter Byass who helped conceptualize this Special Issue, and the Responsible Editor, Dr Jennifer Stewart Williams who carried this forward to fruition.

Prof Karen Hofman

Director Priceless SA

SA MRC/ Wits Centre for Health Economics and Decision Science

Wits School of Public Health, Johannesburg SA

